# In-situ observation of ancient co-fusion steelmaking process based on HT-CLSM

**DOI:** 10.1038/s41598-020-76326-5

**Published:** 2020-11-16

**Authors:** Shangxiao Qiao, Wei Qian

**Affiliations:** Institute of Cultural Heritage and History of Science and Technology, University of Science and Technology Beijing, Beijing, 100083 People’s Republic of China

**Keywords:** Chemistry, Engineering, Materials science, Physics

## Abstract

Observing and analyzing the microstructure of zone samples had been an essential concept in the study of co-fusion steelmaking. High-temperature confocal laser scanning microscope (HT-CLSM) provided a new method in the in-situ observation and real-time analysis of the co-fusion steelmaking process. In this research, a series of experiments had been designed based on Shen Kuo’s *The Dream Pool Essays* (a famous ancient Chinese literature) and carried out by HT-CLSM. The results showed that a new interface was formed with the phase transition during the heating process, which had an essential influence on promoting the carbon diffusion rate in co-fusion steelmaking. The cast iron zone occurred in mushy solidification because of decarbonization. There were collision, aggregation, and other behaviors of inclusions in the cast iron zone and they moved towards the boundary, which led to the purity of the matrix and the aggregation of a large number of inclusions at the boundary.

## Introduction

Because of the long history and universal application, swords represented the highest metallurgy and material processing in history^1^. In a wide range of historical periods, it was a challenge for civilizations to forge swords by smelting the high-quality steel. Different cultures had significant differences in the processes of smelting steel in ancient times, such as the “Wootz” process in India and Sri-Lanka^2,3^ and the “Bulat” process in Central Asia^4^ to smelt crucible steel, the “Zuku-oshi” process to smelt cast iron, and the “Kera-oshi” process to smelt crude steel in Japan^5^. As an essential steelmaking process in ancient China, the co-fusion steelmaking represented the Chinese unique technical tradition based on cast iron.

There were significant differences in iron and steel technology systems between the East and the West in the ancient world. The invention and extension of the cast iron and cast iron steelmaking technology was the core divergence before the fourteenth century CE. There were two main steelmaking processes from cast iron: decarburization and co-fusion, which was the same as the technological thought of the modern steelmaking industry. The term “co-fusion” was first proposed by Needham^6^ in 1955, and it corresponded to smelt medium–high carbon steels from the combination of cast iron and wrought iron. Ancient cast iron mainly contained low content of silicon and sulfur because of charcoal as fuel, which was different from the composition of modern cast iron. Unlike the modern wrought iron, the term “wrought iron” here meant that the carbon content of iron was too low to be steel. It did not contain any information about smelting technology. Co-fusion was mainly used to produce “blade steel,” which was essential for making swords in ancient times. However, the co-fusion process’s technical details have been lost, and the microstructure characteristics of the co-fusion samples were not evident yet.

The research of co-fusion steelmaking was an essential topic in Chinese metallurgical history, which has been discussed by scientists from different angles. Needham^6^, Miao^7^, and He^8^ have carried out simulation experiments to replicate this ancient technology; Ke^9^, Miao^7^, Chen^10^, Jia^11^, and Liu^12^ have analyzed the microstructure of suspected archaeological co-fusion samples; Hua^13^ and Xie^14^ discussed the co-fusion process principle from the philosophy of “harmony.” Although scientists have made some achievements from the perspective of ancient literature, archaeological relics, and simulation experiments, restricted by the limitations of traditional analytical methods, the current understanding of co-fusion mainly stayed in the microstructure analysis of samples at room temperature. However, there is a lack of in-situ study on the physical and chemical changes in the process of high temperature.

High-temperature confocal laser scanning microscope (HT-CLSM) is an optical microscopic observation technology developed at the end of the twentieth century. This technology combines the laser analysis system with the high-temperature observation system, which can observe the surface changes of samples in real-time and save them as video records. At present, it has been widely used in the in-situ observation and research of modern iron and steel materials. The research focuses on phase transformation^15–18^, movement behavior of inclusions^19–21^, and connection of different materials^22–25^. However, the in-situ observation and research of ancient Chinese iron and steel materials have not been reported yet.

In this paper, HT-CLSM was introduced into the study of ancient co-fusion steelmaking for the first time. Based on the technology recorded in ancient literature, the reactions in simulation experiments were observed in-situ, and the real-time dynamic analysis of these reactions have been carried out. With this technology’s help, the high-temperature reactions such as the fusion of cast iron, solidified due to decarburization, the migration of the solid–liquid interface, and the movement of inclusions could be adequately characterized in-situ. Based on the in-situ observation results, on the one hand, the microstructure characteristics of the simulated co-fusion samples could be summarized and compared with the suspected archaeological co-fusion samples; on the other hand, the results provided a reference for the in-depth restoration of ancient co-fusion technology.

## Experimental

### Experimental design basis

The basis of simulation experiment design was crucial. This simulation experiment was designed based on the record of co-fusion steelmaking technology in Shen Kuo’s *The Dream Pool Essays*^26^, a famous ancient Chinese literature, and it was improved under the laboratory conditions. Since ancient Chinese craftsmen usually used charcoal as fuel, and the furnace’s structure was relatively small, so the upper limit of the simulation experiment temperature was set to 1200 °C^27^. “Bending wrought iron, inserting cast iron” meant that the wrought iron was processed into a suitable shape to ensure the formation of a diffusion interface between cast iron and wrought iron. The purpose of “sealing with clay and refining” was to form a relatively independent atmosphere in the smelting process to prevent the raw materials’ oxidation. The simulation experiment’s ideal condition was to use argon as the protective gas in the whole process. “Forging the materials” was intended to make the raw materials tightly by physical forging under suitable temperature. The formation of a solid–liquid interface was more conducive to the migration of carbon atoms between the cast iron zone and the wrought iron zone under the condition of high temperature. Although naked eyes tightly observed the materials in ancient technological conditions, there might be a gap at the interface, so it was reasonable to have a gap of less than 0.1 mm between the cast iron zone and the wrought iron zone in the sample.

### Samples preparation

The physical and chemical changes of raw materials in the simulation experiment were observed in-situ using ultra-high pure white cast iron with trace Si and S and industrial wrought iron as raw materials. The composition and process mainly referred to Han^27^ and Wagner^28^. The chemical composition of raw materials selected in the experiment was shown in Table 1.

**Table 1:** Composition of the material for experiments wt%.

Material	C	Si	Mn	P	S
Cast iron	3.40	≤ 0.05	≤ 0.01	≤ 0.005	0.011
Wrought iron	0.1	≤ 0.005	≤ 0.01	0.006	0.010

**Figure 1:**
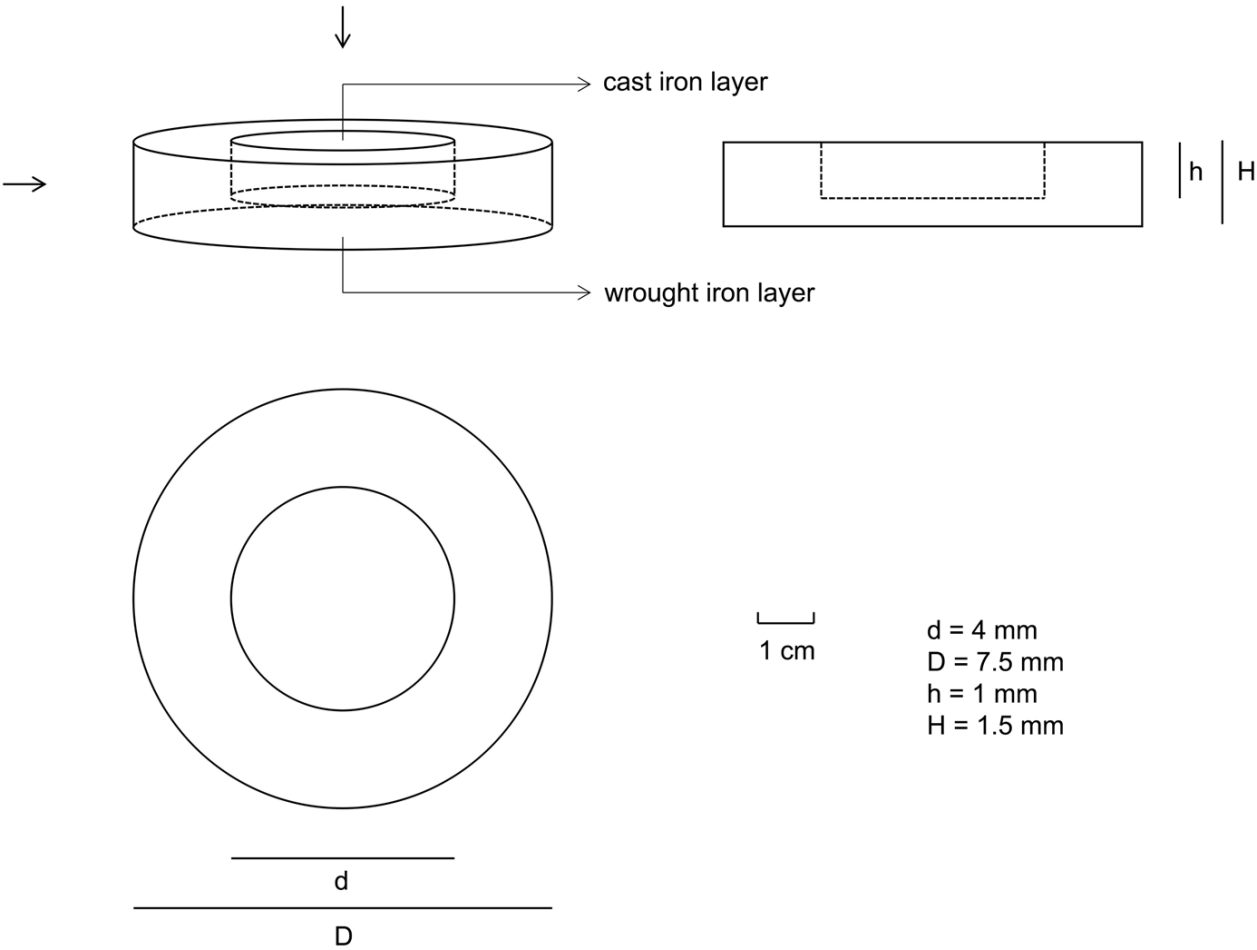
Shape and size of co-fusion sample in simulation experiment.

The cast iron used in the experiment was processed into a cylinder with a diameter of 4 mm and a height of 1 mm, and the wrought iron was processed into a cylinder with a diameter of 7.5 mm and a height of 1.5 mm. Suitable space was pulled out along the centre of the upper surface, and the cast iron was embedded into the wrought iron appropriately. According to the above experimental scheme, the volume of the cast iron was 12.56 mm^3^, and the volume of the wrought iron was 53.67 mm^3^, the ratio of the cast and wrought iron was 1:4.25. The size and shape were as follows in Fig. 1.

### Experimental method

The HT-CLSM used in the experiment was mainly composed of two parts: the laser confocal imaging system and the heating system. The laser confocal imaging system mainly used VL2000DX purple laser, the wavelength was 408 nm, and the resolution could reach 450 nm at high temperature. The heating system could control temperature by the program, and the minimum heating speed was 0.1 °C/s; the maximum heating speed was 300 °C/min. In this experiment, the scanning speed parameters is 15 frames per second and magnification 100 times were recorded automatically.

The prepared sample was cleaned, inlaid, polished, then removed from the inlaid mould and placed into the heating furnace of the high-temperature confocal scanning microscope. Due to the limitation of ancient furnace temperature conditions, the upper limit of the simulation experiment temperature needed to be controlled at 1200 °C^27^. The sample was heated to 1200 °C (at 115 °C/min), kept for 1 h, and cooled down with 130 °C /min cooling rate. Moreover, to prevent the adverse effects of sample oxidation, the sample chamber was filled with high purity Ar as the protective gas. The heating rate mainly refers to the data of the steel ingot placed in the open charcoal furnace during the simulation experiment in a sword workshop. The cooling rate mainly refers to the steel ingot cooling rate in air. The authors agree that argon as a protective atmosphere did not exist in the ancient times. However, the importance of controlling the relatively independent atmosphere is often mentioned in the literature of ancient co-fusion process.

## In-situ observation results

### Phase transformation in the heating process

In theory, several phase transformations were mainly involved in the experimental conditions: the transformation of Fe_3_C (Cementite phase), α (Ferrite phase) to γ (Austenite phase), and part of L (Liquid phase) in the cast iron zone; the transformation to L was not involved in the wrought iron zone; and the phase transformation occurred in the process of carburization and decarburization of two raw materials.

**Figure 2:**
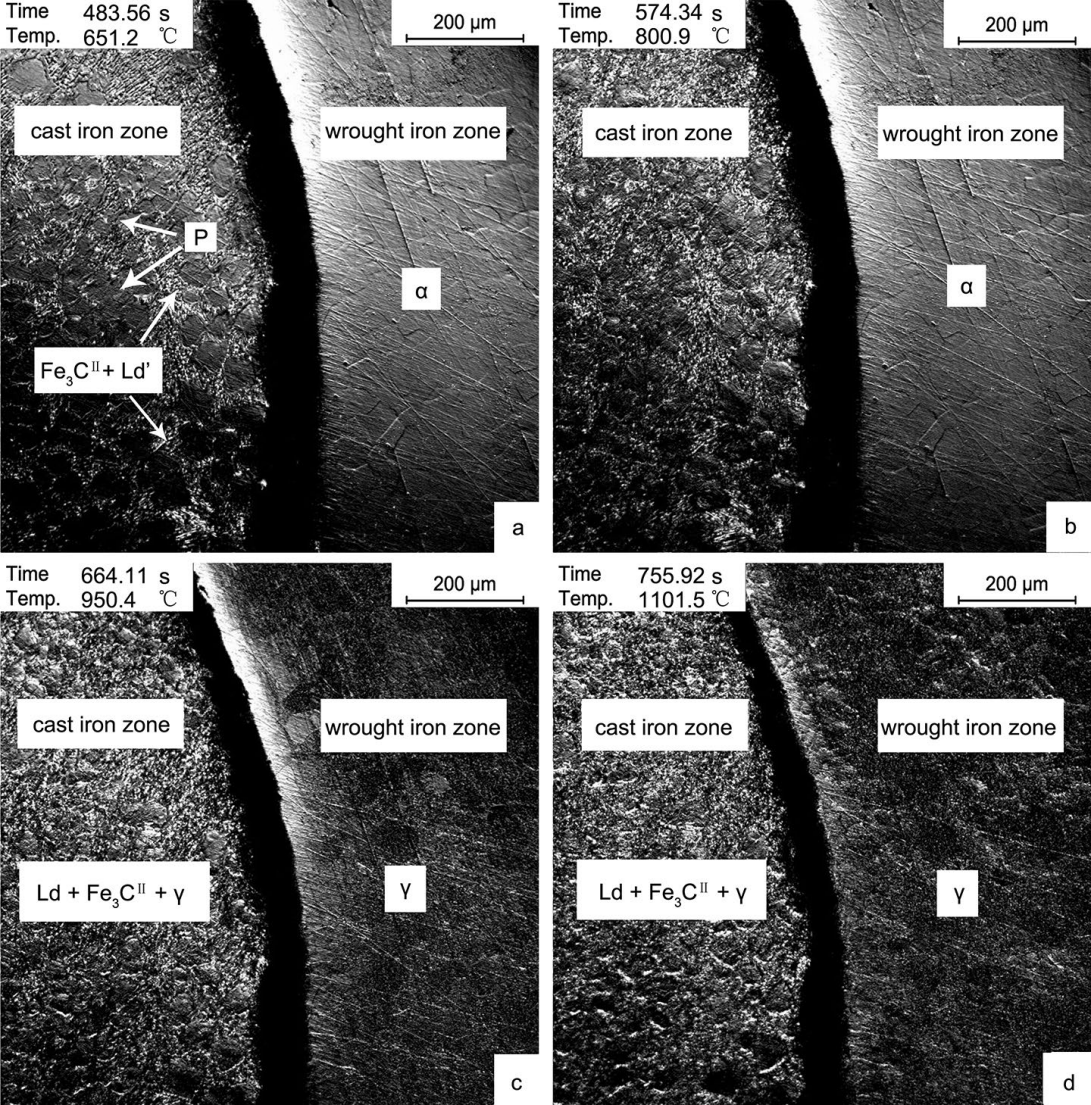
Microstructure transformation in the process of heating to 1100 °C. (**a**) 650 °C; (**b**) 800 °C; (**c**) 950 °C; (**d**) 1100 °C.

When the temperature rose below 1100 °C, the first phase transformation was from α to γ. The microstructure of the cast iron zone was P (Pearlite phase) + Fe_3_C^II^ (Eutectoid Cementite phase) + Ld’ (Modified Ledeburite phase) at room temperature transformed to γ + Fe_3_C^II^ + Ld (Ledeburite phase) with the increase of temperature. The Fe–C phase diagram showed that the transformation temperature was 727 °C, which is slightly higher than the observed temperature. The wrought iron zone was mainly transformed from α to γ. The microstructures of the sample at 650 °C, 800 °C, 950 °C, and 1100 °C were observed in-situ, and the results were shown in Fig. 2.

According to Fig. 2a, the microstructure of the cast iron zone and the wrought iron zone of the sample at 650 °C were mainly the same as that at room temperature, and there was no visible phase transformation. In Fig. 2b, there was no apparent phase transformation at 800 °C in the wrought iron zone, which is related to the low carbon content. In the cast iron zone, Ld’ → Ld, P → γ, part of Fe_3_C^II^ was dissolved in γ. It could be observed that compared with Fig. 2a, the carbides were dissolved. Continue to heat up to 950 °C, as shown in Fig. 2c, α → γ in the wrought iron zone, mainly γ + Fe_3_C^II^ + Ld in the cast iron zone. To 1100 °C, the microstructure of the samples was basically unchanged.

**Figure 3:**
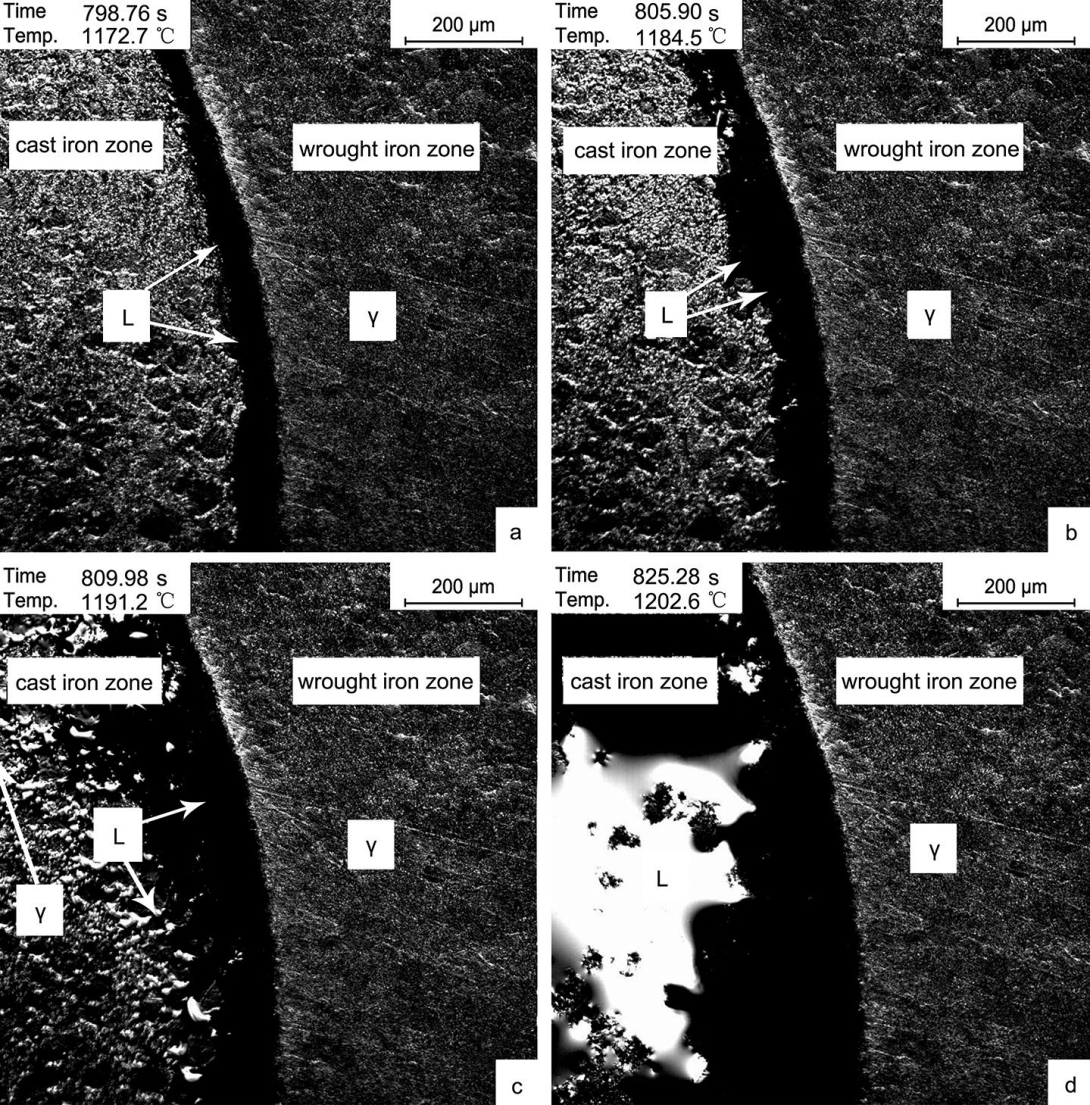
Microstructure transformation after cast iron fused. (**a**) 1170 °C; (**b**) 1180 °C; (**c**) 1190 °C; (**d**) 1200 °C.

Based on the Fe–C phase diagram, the eutectic temperature of cast iron was 1148 °C. Because the expressed temperature should deviate from the actual surface temperature of the sample in the simulation experiment, therefore, attention should be paid when the temperature rose above 1150 °C. When the furnace temperature rose to 1170 °C, the cast iron zone began to fuse, as shown in Fig. 3a. This phenomenon was first seen in the boundary of the cast iron zone. It was rising to 1180 °C, as shown in Fig. 3b, the width of L in the boundary was further increased, and the carbides in the cast iron zone were active. When the temperature was over 1190 °C ,as shown in Fig. 3c, the network carbides in the cast iron zone fused, and the difference between the two phases could be observed, that is, the fused carbides and the internal γ. Heating up to 1200 °C, the carbides in the cast iron zone fused utterly, and the phase transformed to L + γ (Fig. 3d).

### Solidification process

**Figure 4:**
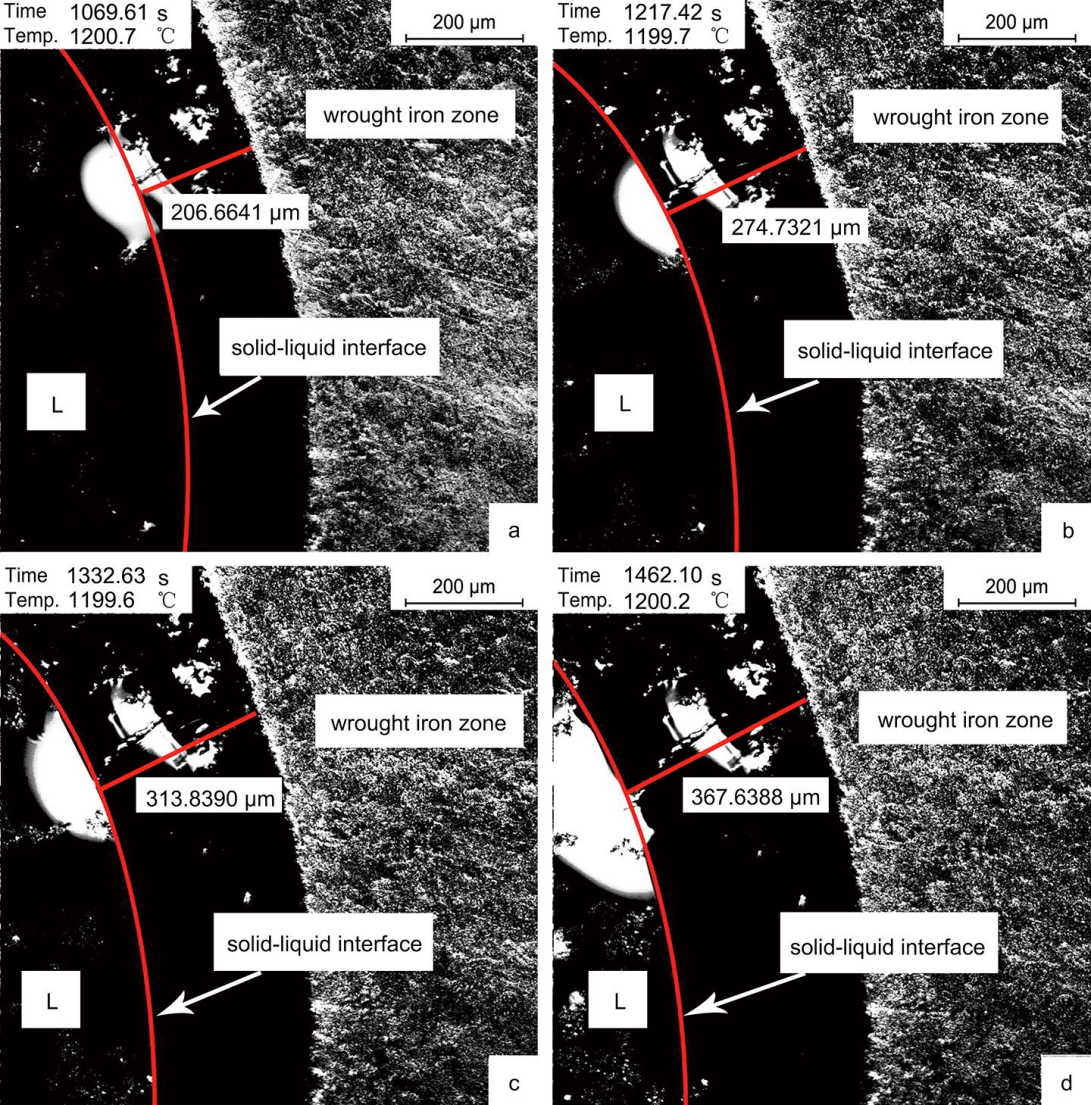
In-situ observation of solid–liquid interface migration at 1200 °C. (**a**) 1069.6 s; (**b**) 1217.4 s; (**c**) 1332.6 s; (**d**) 1462.1 s.

Unlike the solidification with the decrease of temperature, the cast iron zone in the sample solidified due to decarburization under the experimental conditions. After smelting at 1200 °C for 15 min, the in-situ observation was shown in Fig. 4.

**Figure 5:**
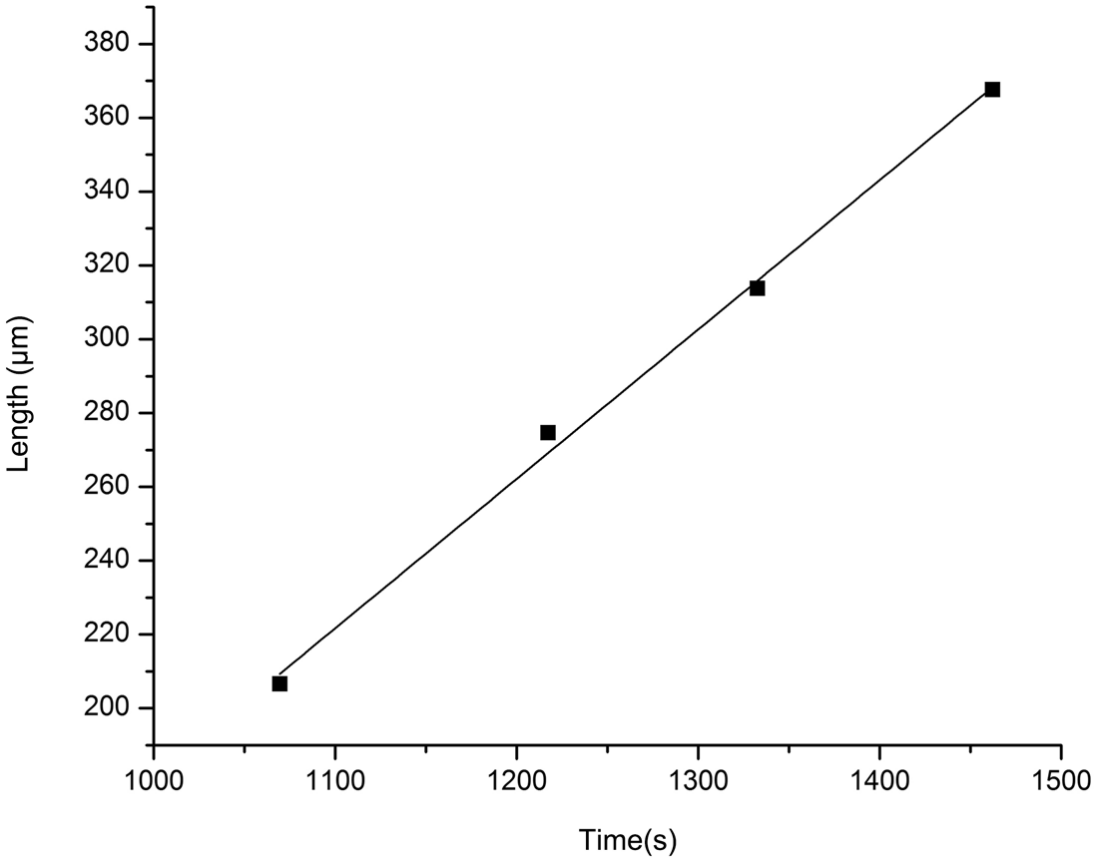
Fitting curve of solid–liquid interface migration rate at 1200 °C.

At 1200 °C, the cast iron zone of solidified slowly due to decarburization. The boundary solidified first to form a transition region, and the carbon content in this region was affected by the diffusion law. Combined with the solidification results in Fig. 4a–d, the size of the solid–liquid interface from the boundary was obtained, and the fitting results could be obtained in Fig. 5.

The results showed a linear relationship between the size of the solid–liquid interface migration and the time. According to Fig. 5, the solid–liquid interface's migration rate was 0.40 μm/s, and the fitting determinable coefficient R^2^ was 0.9955. The migration distance reflected the change of the carbon content at a certain point in the cast iron zone. It was because the solidification mode of the sample was affected by the carbon content. According to the Fe–C phase diagram, when the cast iron zone's carbon content decreased to less than 1.8% at 1200 °C, the sample began to solidify. It could be understood by the diffusion equation solution of a certain length of diffusion couple in the horizontal and vertical directions. Under the given conditions, the solution of the diffusion equation was as follows^29^:

1 $$C\left( {x,t} \right) = \frac{{C_{0} }}{2}\left[ {1 - {\text{erf}}\left( {\frac{x}{{2\sqrt {Dt} }}} \right)} \right]$$

In formula (1), ${\text{erf}}\left( {\frac{x}{{2\sqrt {Dt} }}} \right)$ represented an error function, which could be found out by the particular function table, *x* represented the distance of any point according to the diffusion interface, *t* was the reaction time, *C* was the concentration of any point, in this experiment it was the concentration of carbon. When *C* was given in any direction,

2 $$x^{2} \propto Dt$$

In this plane, the migration distance *ΔL* was directly proportional to *x*^2^, so it could be seen that:

3 $${\Delta }L \propto Dt$$

When the diffusion coefficient *D* was fixed in the simulation experiment, the size of the solid–liquid interface from the boundary had a linear relationship with the time, which was consistent with the experimental results.

### Aggregation and movement behaviour of inclusions

Unlike the term “inclusions” in the modern steel industry, inclusions in ancient steels were quite different in size, number, and composition. Due to the control of various parameters in the ancient steel smelting process cannot be compared with that in modern times. In this experiment, even high purity Ar gas was maintained during the observation. The surface oxidation is unavoidable. Therefore, the appearance and movement of inclusions in the simulation sample caused by oxidation were essential for analyzing the microstructure of co-fusion artifacts.

**Figure 6:**
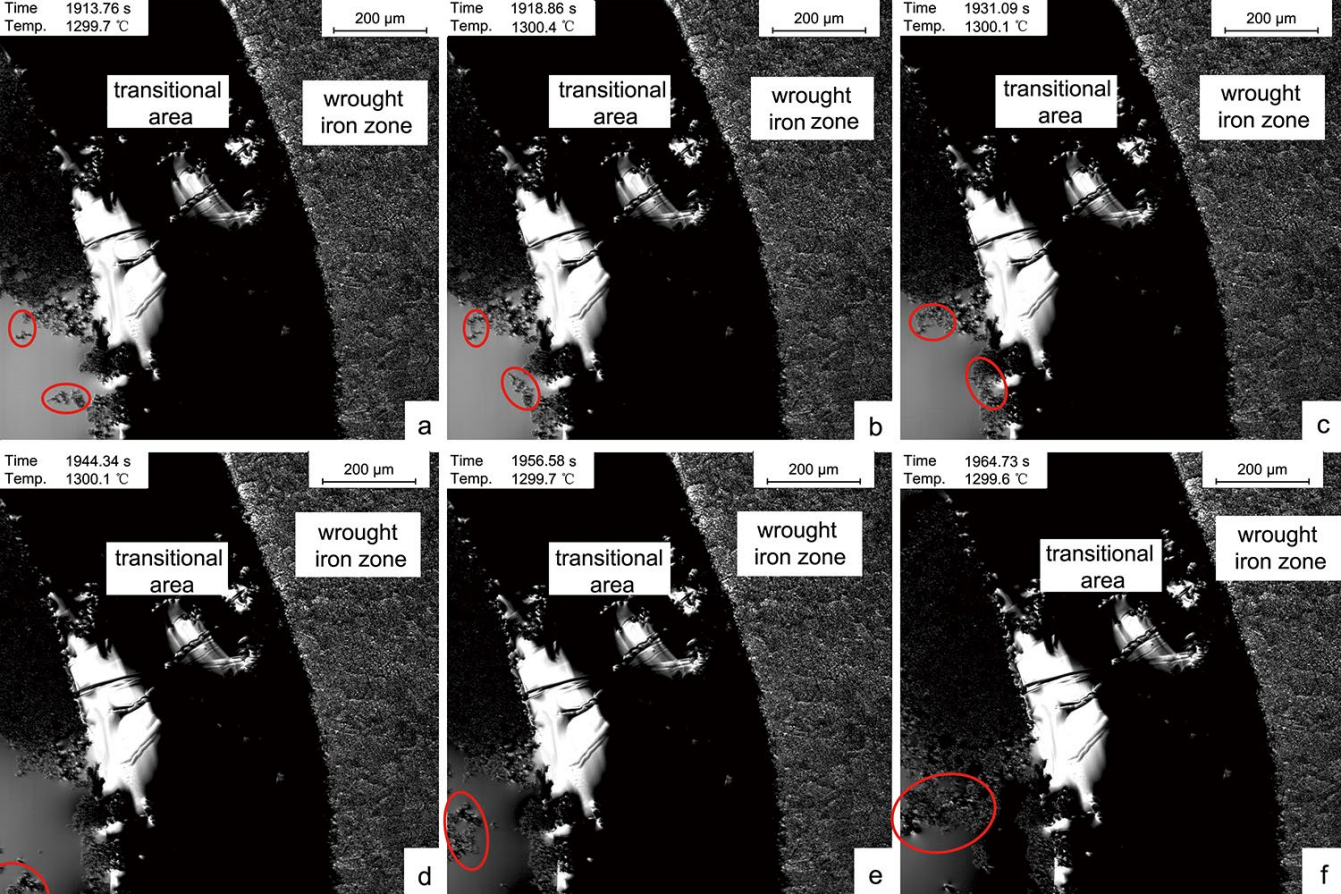
Aggregation and movement behaviour of inclusions. (**a**) 1913.7 s; (**b**) 1918.8 s; (**c**) 1931.0 s; (**d**) 1944.3 s; (**e**) 1956.5 s; (**f**) 1964.7 s.

In-situ observation of the aggregation and movement behavior of inclusions in the co-fusion process was helpful to understand the size, shape, and distribution of inclusions in the archaeological co-fusion samples at room temperature. Because of the cast iron zone fused, the inclusions floated, aggregated, and migrated in the simulation experiment. The results were shown in Fig. 6.

According to Fig. 6a–c, the small-sized inclusions in the cast iron zone initially formed large-sized inclusions after collision and aggregation, then moved to the solid–liquid interface under the action of liquid surface tension. The phenomenon of inclusions aggregation began to appear. In Fig. 6d–f, the inclusions aggregated near the center of the cast iron zone, then moved to the solid–liquid interface. With the continuous outward movement of inclusions in the cast iron zone, there were few inclusions in the center, and the matrix was pure. However, the inclusions at the boundary would gather in large quantities.

## Analysis and discussion

### Analysis of in-situ observation results

The in-situ observation and analysis of the simulated co-fusion samples during the heating process revealed that when the temperature was lower than the fusing point of cast iron, the solid–gas–solid interface was formed. The carbon atoms in the cast iron zone were affected by temperature and interface, and the diffusion rate was slow. As the temperature rose to the liquid phase transformation of carbides in cast iron, the boundary of the cast iron zone fused first. The solid–liquid interface was formed between the zones, which reduced the free energy of the whole system^30^, and increased the diffusion rate of carbon atoms from the cast iron zone to the wrought iron zone. Therefore, it could be seen that during the heating process, a new interface had been formed between the zones with the phase transformation, which had a significant influence on improving the carbon diffusion rate in co-fusion. The diffusion connection and new solid–liquid interface formation were essential features in the co-fusion process of heating.

Based on the convection and diffusion theory, the composition of the transition area would undergo complex changes after diffusion. In ideal conditions, the reaction–diffusion rate constant at the interface was equivalent to the surface reaction rate constant, which was shown as the surface reaction law^31^. Under the condition of the same concentration difference and liquid–solid interface, the higher the temperature was, the faster the diffusion rate from the liquid phase to the transition area was, and the faster the transition area diffused to the wrought iron zone was. After the formation of the new interface, there was a relatively high degree of carbonization gradient between the zones under the conditions of temperature, interface, and composition. The carbon atoms diffused rapidly from the cast iron zone to the wrought iron zone. The decrease of carbon atom concentration at the cast iron zone resulted in the rapid solidification of the transition area.

For inclusions, there were observable behaviors of precipitation, collision, and movement in the cast iron zone. The inclusions near the center precipitated again and moved towards the boundary, which made the matrix pure. There were a massive number of inclusions and shrinkage cavities at the boundary. No visible movement behavior of inclusions was observed in the wrought iron zone. The difference of inclusions size and quantity between the center and boundary of the cast iron zone, and this feature was not found in the wrought iron zone, which was a vital microstructure feature of co-fusion samples.

### Microstructure of ancient co-fusion samples from the results of simulation experiments

**Figure 7:**
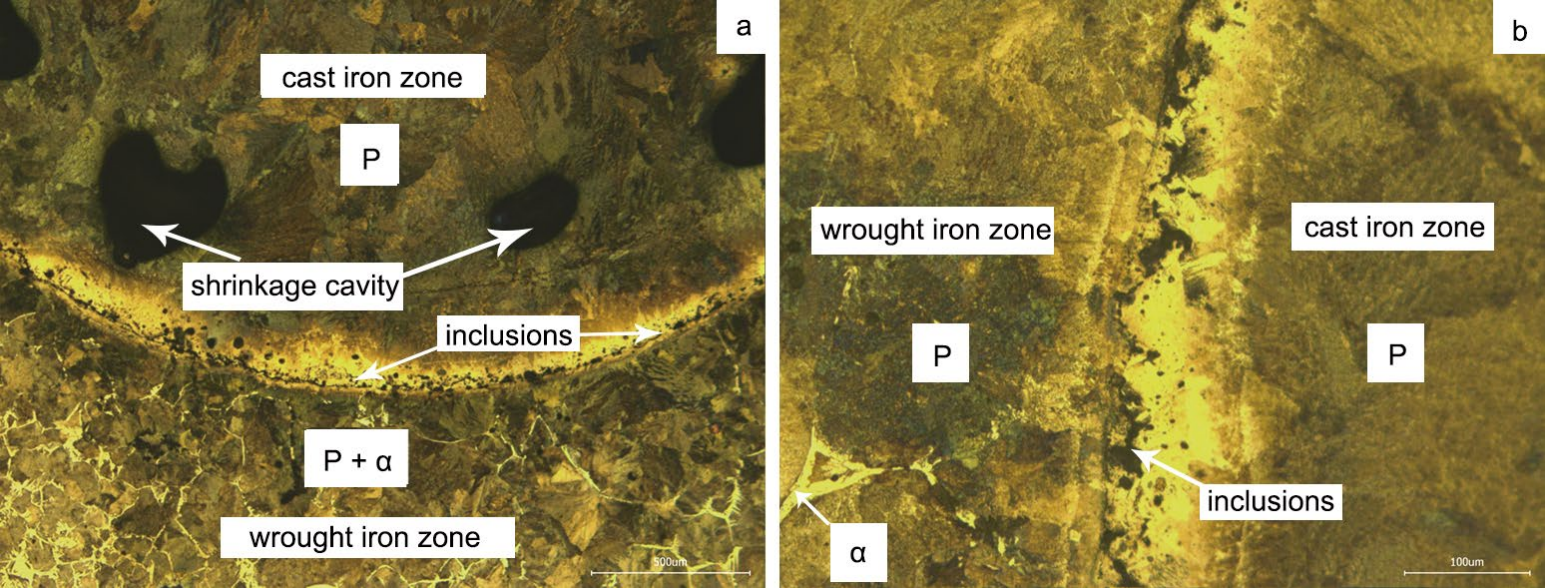
The metallographic microstructure of the simulated co-fusion sample. (**a**) × 50; (**b**) × 200.

The analysis and determination of microstructure characteristics of the ancient co-fusion samples were one of the critical problems in Chinese metallurgical history. In-situ observation results might provide new evidence for the problem. Based on the metallographic analysis results of the samples at room temperature, as shown in Fig. 7, it was discussed from the aspects of microstructure and inclusions.

From the perspective of microstructures, the in-situ observation results of the co-fusion sample showed that there was a new interface between the zones, which significantly promoted the carbon diffusion rate. With the diffusion of carbon from cast iron to wrought iron, the cast iron zone solidified due to decarburization. Because of the same process transformation in different regions, the final microstructure of the sample was more determined by the initial state. The carbon concentration gradient might exist in different areas after diffusion for a while, and there might be an evident carbon diffusion phenomenon at the interface of zones. Based on the analysis results, the study on the microstructure of ancient co-fusion samples should focus on the carbon content difference and the phenomenon of carbon diffusion in different regions.

From the perspective of inclusions, there was a significant difference between the cast iron zone and the wrought iron zone in co-fusion. The simulation experiment results showed that the inclusions tend to move towards the boundary after aggregation due to fusion and decarburization of the cast iron zone, while the matrix was quite pure; this phenomenon was not observed in the wrought iron zone. It was suggested that the size and distribution of inclusions in different regions might be significantly different. First of all, unlike modern cast iron and wrought iron, the composition, size, and quantity of inclusions in ancient were quite different^25^. Secondly, there were further changes of inclusions in co-fusion, mainly the inclusions in the cast iron zone gather towards the boundary, and the matrix was pure, and further forging would extrude the inclusions at the boundary. There was no visible movement behavior of inclusions in the wrought iron zone, but the solubility of elements would be different due to the change of carbon content, then there were differences in number, size, and shape.

Combined with the micro-analysis of iron artifacts in previous studies, the results of simulation experiments could well correspond with the characteristics of archaeological co-fusion samples. In terms of microstructures, archaeological co-fusion samples contained many characteristics related to cast iron^9–12,32^. However, the results of simulation experiments showed the characteristics of homogenization. It was due to the long holding time under high temperatures in the simulation experiments, but the archaeological samples might have a short time of heat preservation. On the other hand, the diffusion phenomenon between the interfaces was quite evident. In terms of inclusions, they were rare in the matrix of cast iron zone and distributed dispersedly in the wrought iron zone. Furthermore, generous inclusions were concentrated at the interface. This phenomenon could be seen in both archaeological artifacts and simulation experiment samples.

## Conclusions

1. The high-temperature laser confocal scanning microscopy could be used for in-situ observation to understand the co-fusion steelmaking process's physical and chemical changes. It had great potential in real-time analysis of the phase transformation in heating, the formation of new phases in solidification, the aggregation and migration of inclusions. Combined with the analysis results of simulated co-fusion samples at room temperature, the micro-characteristics of ancient co-fusion samples could be discussed.
2. In the heating process, a new solid–liquid interface was formed between the cast iron zone and the wrought iron zone, which significantly influenced the carbon diffusion rate. With the diffusion of carbon, the new solid–liquid interface gradually migrated to the center at a specific rate. The final solidification mode was mushy solidification. The inclusions in the cast iron zone floated, aggregated, and migrated to the solid–liquid interface. As a result, the cast iron zone matrix was pure, and quantity inclusions gathered at the boundary. The in-situ observation results provided an essential basis for further restoration of ancient co-fusion steelmaking technology.

## Data Availability

The data is available in form of Excel files from qiaoshangxiao115@163.com on e-mail request.
